# 4-Meth­oxy-*N*′-(3-nitro­benzyl­idene)benzohydrazide

**DOI:** 10.1107/S1600536812020454

**Published:** 2012-05-16

**Authors:** Jin-Long Hou, Ye Bi

**Affiliations:** a College of Chemistry and Chemical Engineering, Qiqihar University, Qiqihar 161006, People’s Republic of China

## Abstract

In the title compound, C_15_H_13_N_3_O_4_, the dihedral angle between the benzene rings is 3.1 (3)°. The mol­ecule displays an *E* conformation about the C=N bond. In the crystal, mol­ecules are linked *via* N—H⋯O hydrogen bonds, generating chains that propagate along the *b*-axis direction. There is also a C—H⋯O inter­action present.

## Related literature

For the biological properties of hydrazone compounds, see: Cukurovali *et al.* (2006 ▶); Karthikeyan *et al.* (2006 ▶); Kucukguzel *et al.* (2006 ▶). For related hydrazone compounds, see: Hou (2009 ▶, 2012 ▶); Mohd Lair *et al.* (2009 ▶); Fun *et al.* (2008 ▶); Zhang *et al.* (2009 ▶); Khaledi *et al.* (2008 ▶). For standard bond lengths, see: Allen *et al.* (1987 ▶).
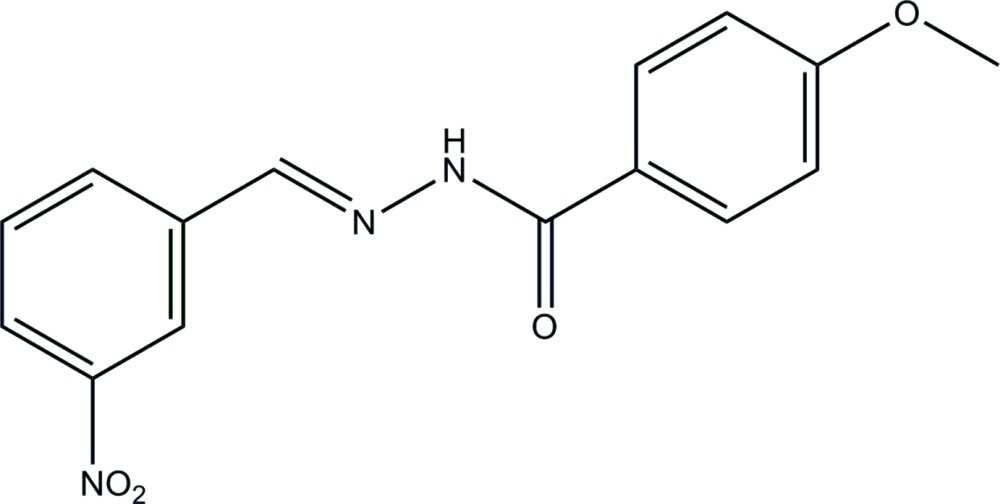


## Experimental

### 

#### Crystal data

C_15_H_13_N_3_O_4_
*M* *_r_* = 299.28
Monoclinic, 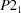

*a* = 6.8472 (17) Å
*b* = 4.8269 (16) Å
*c* = 21.414 (3) Å
β = 96.696 (2)°
*V* = 702.9 (3) Å^3^
*Z* = 2
Mo *K*α radiation
μ = 0.11 mm^−1^
*T* = 298 K
0.17 × 0.13 × 0.13 mm

#### Data collection

Bruker SMART 1000 CCD area-detector diffractometer
Absorption correction: multi-scan (*SADABS*; Sheldrick, 1996 ▶) *T*_min_ = 0.982, *T*_max_ = 0.987
3686 measured reflections
1445 independent reflections
1275 reflections with *I* > 2σ(*I*)
*R*_int_ = 0.036

#### Refinement

*R*[*F* ^2^ > 2σ(*F* ^2^)] = 0.034
*wR*(*F* ^2^) = 0.085
*S* = 1.05
1445 reflections
203 parameters
2 restraints
H atoms treated by a mixture of independent and constrained refinement
Δρ_max_ = 0.11 e Å^−3^
Δρ_min_ = −0.16 e Å^−3^

### 

Data collection: *SMART* (Bruker, 1998 ▶); cell refinement: *SAINT* (Bruker, 1998 ▶); data reduction: *SAINT*; program(s) used to solve structure: *SHELXS97* (Sheldrick, 2008 ▶); program(s) used to refine structure: *SHELXL97* (Sheldrick, 2008 ▶); molecular graphics: *SHELXTL* (Sheldrick, 2008 ▶); software used to prepare material for publication: *SHELXTL*.

## Supplementary Material

Crystal structure: contains datablock(s) global, I. DOI: 10.1107/S1600536812020454/su2422sup1.cif

Structure factors: contains datablock(s) I. DOI: 10.1107/S1600536812020454/su2422Isup2.hkl

Supplementary material file. DOI: 10.1107/S1600536812020454/su2422Isup3.cml

Additional supplementary materials:  crystallographic information; 3D view; checkCIF report

## Figures and Tables

**Table 1:** Hydrogen-bond geometry (Å, °)

*D*—H⋯*A*	*D*—H	H⋯*A*	*D*⋯*A*	*D*—H⋯*A*
N3—H3⋯O3^i^	0.90 (2)	2.03 (2)	2.861 (3)	154 (3)
C6—H6⋯O1^ii^	0.93	2.60	3.268 (3)	129

Symmetry codes: (i) 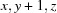; (ii) 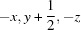.

**Fig. 1.**
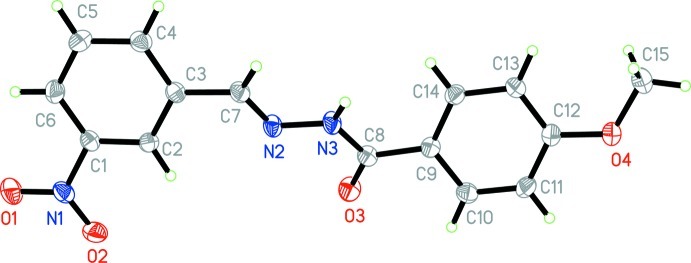
The molecular structure of the title molecule, with the atom numbering. The displacement ellipsoids are drawn at 30% probability level.

**Fig. 2.**
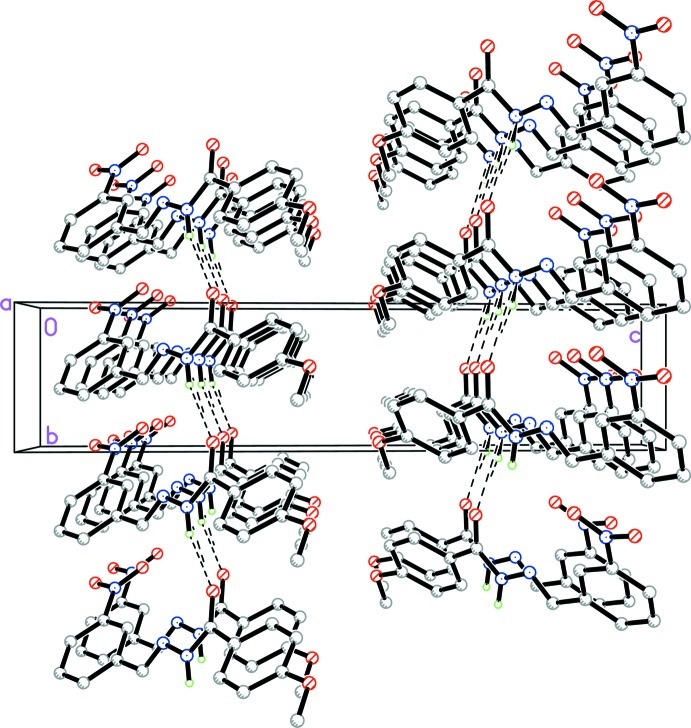
A view along the a axis of the crystal packing of the title compound, with the N-H···O hydrogen bonds shown as dashed lines (see Table 1 for details).

## supplementary crystallographic information

### Comment

Hydrazones derived from the condensation reactions of hydrazides with aldehydes show excellent biological properties (Cukurovali *et al.*, 2006; Karthikeyan *et al.*, 2006; Kucukguzel *et al.*, 2006). In the last few years, the crystal structure of a large number of hydrazone compounds have been reported (Hou, 2009; Hou, 2012; Lair *et al.*, 2009; Fun *et al.*, 2008; Zhang *et al.*, 2009; Khaledi *et al.*, 2008). Herein we report on the synthesis and crystal structure of the title compound, derived from the condensation reaction of 3-nitrobenzaldehyde and 4-methoxybenzohydrazide.

The molecular structure of the title compound is shown in Fig. 1. The dihedral angle between the two benzene rings is 3.1 (3)°. The molecule displays an *E* conformation about the C═N bond. All the bond lengths are within normal ranges (Allen *et al.*, 1987).

In the crystal, molecules are linked *via* N–H···O hydrogen bonds (Table 1) generating chains along the *b* axis direction (Fig. 2). There is also a C-H···O interaction present (Table 1).

### Experimental

3-Nitrobenzaldehyde (1.0 mmol, 151 mg) and 4-methoxybenzohydrazide (1.0 mmol, 166 mg) were mixed and refluxed with stirring for two hours. Yellow single crystals were formed after slow evaporation of the solution in air for a week.

### Refinement

The NH H atom was located in a difference Fourier map and refined with the N–H distance restrained to 0.90 (2) Å and *U*_iso_(H) = 0.08 Å^2^. The other H atoms were placed in calculated positions and constrained to ride on their parent atoms: C–H = 0.93 and 0.96 Å for CH and CH_3_ H atoms, respectively, with *U*_iso_(H) = k × *U*_eq_(C), where k = 1.5 for CH_3_ H atoms, and 1.2 for other H atoms. In the final cycles of refinement, in the absence of significant anomalous scattering effects, 929 Friedel pairs were merged and \Df " set to zero.

### Crystal data

**Table:**

C_15_H_13_N_3_O_4_	*F*(000) = 312
*M**_r_* = 299.28	*D*_x_ = 1.414 Mg m^−^^3^
Monoclinic, *P*2_1_	Mo *K*α radiation, λ = 0.71073 Å
Hall symbol: P 2yb	Cell parameters from 1829 reflections
*a* = 6.8472 (17) Å	θ = 2.8–27.0°
*b* = 4.8269 (16) Å	µ = 0.11 mm^−^^1^
*c* = 21.414 (3) Å	*T* = 298 K
β = 96.696 (2)°	Block, yellow
*V* = 702.9 (3) Å^3^	0.17 × 0.13 × 0.13 mm
*Z* = 2	

### Data collection

**Table:**

Bruker SMART 1000 CCD area-detector diffractometer	1445 independent reflections
Radiation source: fine-focus sealed tube	1275 reflections with *I* > 2σ(*I*)
Graphite monochromator	*R*_int_ = 0.036
ω scans	θ_max_ = 25.5°, θ_min_ = 2.9°
Absorption correction: multi-scan (*SADABS*; Sheldrick, 1996)	*h* = −7→8
*T*_min_ = 0.982, *T*_max_ = 0.987	*k* = −5→5
3686 measured reflections	*l* = −25→24

### Refinement

**Table:**

Refinement on *F*^2^	Primary atom site location: structure-invariant direct methods
Least-squares matrix: full	Secondary atom site location: difference Fourier map
*R*[*F*^2^ > 2σ(*F*^2^)] = 0.034	Hydrogen site location: inferred from neighbouring sites
*wR*(*F*^2^) = 0.085	H atoms treated by a mixture of independent and constrained refinement
*S* = 1.05	*w* = 1/[σ^2^(*F*_o_^2^) + (0.0405*P*)^2^ + 0.0855*P*] where *P* = (*F*_o_^2^ + 2*F*_c_^2^)/3
1445 reflections	(Δ/σ)_max_ = 0.001
203 parameters	Δρ_max_ = 0.11 e Å^−^^3^
2 restraints	Δρ_min_ = −0.16 e Å^−^^3^

### Special details

**Table:**

Geometry. Bond distances, angles etc. have been calculated using the rounded fractional coordinates. All su's are estimated from the variances of the (full) variance-covariance matrix. The cell esds are taken into account in the estimation of distances, angles and torsion angles
Refinement. Refinement of *F*^2^ against ALL reflections. The weighted *R*-factor *wR* and goodness of fit *S* are based on *F*^2^, conventional *R*-factors *R* are based on *F*, with *F* set to zero for negative *F*^2^. The threshold expression of *F*^2^ > σ(*F*^2^) is used only for calculating *R*-factors(gt) *etc*. and is not relevant to the choice of reflections for refinement. *R*-factors based on *F*^2^ are statistically about twice as large as those based on *F*, and *R*- factors based on ALL data will be even larger.

### Fractional atomic coordinates and isotropic or equivalent isotropic displacement parameters (Å^2^)

**Table:**

	*x*	*y*	*z*	*U*_iso_*/*U*_eq_	
O1	−0.0350 (3)	−0.0065 (5)	0.08818 (9)	0.0671 (9)	
O2	0.1567 (3)	−0.1359 (5)	0.16985 (9)	0.0556 (7)	
O3	0.9986 (3)	−0.0624 (4)	0.29825 (8)	0.0492 (6)	
O4	1.7677 (3)	0.4319 (5)	0.44850 (8)	0.0531 (6)	
N1	0.1167 (3)	0.0136 (5)	0.12433 (10)	0.0439 (7)	
N2	0.8115 (3)	0.3103 (4)	0.21877 (9)	0.0376 (7)	
N3	0.9792 (3)	0.3715 (5)	0.25841 (10)	0.0393 (6)	
C1	0.2599 (3)	0.2248 (5)	0.11108 (11)	0.0352 (7)	
C2	0.4255 (3)	0.2586 (5)	0.15295 (11)	0.0347 (7)	
C3	0.5608 (3)	0.4617 (5)	0.14098 (10)	0.0350 (7)	
C4	0.5242 (4)	0.6221 (6)	0.08719 (11)	0.0412 (8)	
C5	0.3576 (4)	0.5802 (6)	0.04504 (12)	0.0454 (9)	
C6	0.2229 (4)	0.3800 (7)	0.05691 (11)	0.0431 (8)	
C7	0.7391 (3)	0.5089 (5)	0.18495 (11)	0.0381 (8)	
C8	1.0667 (4)	0.1711 (5)	0.29592 (11)	0.0362 (8)	
C9	1.2529 (3)	0.2545 (5)	0.33428 (11)	0.0351 (7)	
C10	1.3057 (4)	0.1192 (6)	0.39120 (12)	0.0451 (9)	
C11	1.4763 (4)	0.1874 (6)	0.42782 (11)	0.0460 (9)	
C12	1.6014 (3)	0.3851 (6)	0.40846 (10)	0.0377 (8)	
C13	1.5529 (4)	0.5197 (6)	0.35165 (11)	0.0407 (8)	
C14	1.3780 (4)	0.4556 (6)	0.31541 (11)	0.0388 (8)	
C15	1.8987 (4)	0.6411 (7)	0.43234 (14)	0.0561 (10)	
H2	0.44710	0.14800	0.18870	0.0420*	
H3	1.015 (5)	0.550 (3)	0.2629 (16)	0.0800*	
H4	0.61290	0.76010	0.07930	0.0490*	
H5	0.33640	0.68700	0.00870	0.0550*	
H6	0.11010	0.35040	0.02910	0.0520*	
H7	0.79820	0.68260	0.18790	0.0460*	
H10	1.22470	−0.01830	0.40440	0.0540*	
H11	1.50810	0.09920	0.46630	0.0550*	
H13	1.63710	0.65190	0.33800	0.0490*	
H14	1.34380	0.54920	0.27780	0.0470*	
H15A	1.94650	0.59410	0.39330	0.0840*	
H15B	2.00750	0.65530	0.46480	0.0840*	
H15C	1.83080	0.81520	0.42800	0.0840*	

### Atomic displacement parameters (Å^2^)

**Table:**

	*U*^11^	*U*^22^	*U*^33^	*U*^12^	*U*^13^	*U*^23^
O1	0.0447 (11)	0.091 (2)	0.0624 (12)	−0.0261 (12)	−0.0075 (9)	−0.0027 (13)
O2	0.0545 (11)	0.0582 (14)	0.0551 (11)	−0.0166 (11)	0.0106 (9)	0.0077 (11)
O3	0.0609 (12)	0.0281 (10)	0.0556 (10)	−0.0084 (9)	−0.0057 (9)	−0.0020 (9)
O4	0.0444 (10)	0.0673 (14)	0.0447 (9)	−0.0134 (10)	−0.0075 (8)	0.0069 (10)
N1	0.0373 (12)	0.0506 (15)	0.0445 (11)	−0.0108 (11)	0.0074 (10)	−0.0085 (11)
N2	0.0365 (11)	0.0324 (12)	0.0422 (11)	−0.0062 (9)	−0.0031 (9)	−0.0048 (10)
N3	0.0386 (11)	0.0299 (11)	0.0465 (11)	−0.0082 (10)	−0.0070 (9)	−0.0017 (10)
C1	0.0323 (12)	0.0338 (14)	0.0396 (12)	−0.0023 (10)	0.0050 (10)	−0.0061 (11)
C2	0.0373 (13)	0.0338 (13)	0.0329 (11)	−0.0031 (11)	0.0038 (10)	−0.0011 (10)
C3	0.0341 (12)	0.0325 (14)	0.0384 (12)	−0.0023 (10)	0.0039 (10)	−0.0033 (11)
C4	0.0411 (14)	0.0357 (15)	0.0471 (14)	−0.0045 (12)	0.0063 (11)	0.0016 (12)
C5	0.0533 (16)	0.0439 (17)	0.0384 (13)	0.0015 (13)	0.0023 (12)	0.0059 (13)
C6	0.0388 (13)	0.0488 (17)	0.0400 (12)	−0.0012 (13)	−0.0022 (10)	−0.0042 (13)
C7	0.0372 (13)	0.0324 (14)	0.0439 (13)	−0.0083 (11)	0.0010 (11)	−0.0007 (12)
C8	0.0416 (14)	0.0266 (14)	0.0402 (13)	−0.0029 (11)	0.0037 (11)	−0.0050 (11)
C9	0.0389 (13)	0.0288 (13)	0.0370 (12)	0.0004 (11)	0.0014 (10)	−0.0010 (11)
C10	0.0470 (15)	0.0383 (15)	0.0494 (15)	−0.0091 (13)	0.0027 (12)	0.0078 (12)
C11	0.0510 (16)	0.0481 (17)	0.0369 (13)	−0.0042 (13)	−0.0037 (12)	0.0095 (13)
C12	0.0347 (12)	0.0406 (15)	0.0369 (12)	0.0018 (12)	0.0008 (10)	−0.0042 (12)
C13	0.0382 (13)	0.0410 (16)	0.0425 (13)	−0.0079 (12)	0.0031 (11)	0.0031 (12)
C14	0.0424 (14)	0.0376 (15)	0.0355 (12)	−0.0005 (12)	0.0004 (10)	0.0042 (12)
C15	0.0434 (15)	0.062 (2)	0.0604 (17)	−0.0112 (15)	−0.0041 (13)	0.0008 (16)

### Geometric parameters (Å, º)

**Table:**

O1—N1	1.225 (3)	C9—C14	1.386 (4)
O2—N1	1.218 (3)	C9—C10	1.393 (4)
O3—C8	1.223 (3)	C10—C11	1.369 (4)
O4—C12	1.362 (3)	C11—C12	1.378 (4)
O4—C15	1.420 (4)	C12—C13	1.385 (3)
N1—C1	1.465 (3)	C13—C14	1.384 (4)
N2—N3	1.378 (3)	C2—H2	0.9300
N2—C7	1.267 (3)	C4—H4	0.9300
N3—C8	1.352 (3)	C5—H5	0.9300
N3—H3	0.898 (18)	C6—H6	0.9300
C1—C6	1.379 (4)	C7—H7	0.9300
C1—C2	1.371 (3)	C10—H10	0.9300
C2—C3	1.393 (3)	C11—H11	0.9300
C3—C4	1.386 (3)	C13—H13	0.9300
C3—C7	1.470 (3)	C14—H14	0.9300
C4—C5	1.385 (4)	C15—H15A	0.9600
C5—C6	1.380 (4)	C15—H15B	0.9600
C8—C9	1.489 (3)	C15—H15C	0.9600
			
C12—O4—C15	118.0 (2)	O4—C12—C11	115.4 (2)
O1—N1—O2	123.6 (2)	C11—C12—C13	119.7 (2)
O1—N1—C1	118.1 (2)	C12—C13—C14	119.4 (2)
O2—N1—C1	118.3 (2)	C9—C14—C13	121.1 (2)
N3—N2—C7	115.6 (2)	C1—C2—H2	121.00
N2—N3—C8	119.5 (2)	C3—C2—H2	121.00
C8—N3—H3	122 (2)	C3—C4—H4	119.00
N2—N3—H3	118 (2)	C5—C4—H4	119.00
N1—C1—C6	118.7 (2)	C4—C5—H5	120.00
N1—C1—C2	118.5 (2)	C6—C5—H5	120.00
C2—C1—C6	122.8 (2)	C1—C6—H6	121.00
C1—C2—C3	118.7 (2)	C5—C6—H6	121.00
C2—C3—C7	120.8 (2)	N2—C7—H7	120.00
C2—C3—C4	119.1 (2)	C3—C7—H7	120.00
C4—C3—C7	120.1 (2)	C9—C10—H10	120.00
C3—C4—C5	121.0 (2)	C11—C10—H10	120.00
C4—C5—C6	120.0 (2)	C10—C11—H11	120.00
C1—C6—C5	118.3 (2)	C12—C11—H11	120.00
N2—C7—C3	119.3 (2)	C12—C13—H13	120.00
O3—C8—C9	122.2 (2)	C14—C13—H13	120.00
N3—C8—C9	115.2 (2)	C9—C14—H14	119.00
O3—C8—N3	122.6 (2)	C13—C14—H14	119.00
C10—C9—C14	118.5 (2)	O4—C15—H15A	109.00
C8—C9—C10	118.3 (2)	O4—C15—H15B	109.00
C8—C9—C14	123.2 (2)	O4—C15—H15C	109.00
C9—C10—C11	120.4 (2)	H15A—C15—H15B	109.00
C10—C11—C12	120.9 (2)	H15A—C15—H15C	109.00
O4—C12—C13	124.9 (2)	H15B—C15—H15C	109.00
			
C15—O4—C12—C11	−177.6 (2)	C7—C3—C4—C5	179.7 (2)
C15—O4—C12—C13	2.4 (4)	C2—C3—C4—C5	−1.0 (4)
O2—N1—C1—C2	−4.8 (3)	C3—C4—C5—C6	1.3 (4)
O1—N1—C1—C6	−3.6 (3)	C4—C5—C6—C1	−0.4 (4)
O2—N1—C1—C6	174.9 (2)	O3—C8—C9—C10	−27.8 (4)
O1—N1—C1—C2	176.7 (2)	N3—C8—C9—C14	−29.2 (3)
N3—N2—C7—C3	179.63 (19)	O3—C8—C9—C14	150.7 (3)
C7—N2—N3—C8	−179.7 (2)	N3—C8—C9—C10	152.3 (2)
N2—N3—C8—C9	176.7 (2)	C8—C9—C14—C13	−177.8 (2)
N2—N3—C8—O3	−3.1 (4)	C10—C9—C14—C13	0.7 (4)
N1—C1—C6—C5	179.4 (2)	C8—C9—C10—C11	179.5 (2)
C2—C1—C6—C5	−0.9 (4)	C14—C9—C10—C11	0.9 (4)
C6—C1—C2—C3	1.2 (4)	C9—C10—C11—C12	−1.8 (4)
N1—C1—C2—C3	−179.2 (2)	C10—C11—C12—C13	0.9 (4)
C1—C2—C3—C4	−0.2 (3)	C10—C11—C12—O4	−179.1 (2)
C1—C2—C3—C7	179.0 (2)	O4—C12—C13—C14	−179.3 (3)
C2—C3—C7—N2	27.7 (3)	C11—C12—C13—C14	0.7 (4)
C4—C3—C7—N2	−153.1 (2)	C12—C13—C14—C9	−1.6 (4)

### Hydrogen-bond geometry (Å, º)

**Table:**

*D*—H···*A*	*D*—H	H···*A*	*D*···*A*	*D*—H···*A*
N3—H3···O3^i^	0.90 (2)	2.03 (2)	2.861 (3)	154 (3)
C6—H6···O1^ii^	0.93	2.60	3.268 (3)	129

Symmetry codes: (i) *x*, *y*+1, *z*; (ii) −*x*, *y*+1/2, −*z*.
